# Antimicrobial resistance and the COVID-19 pandemic

**DOI:** 10.2471/BLT.21.287752

**Published:** 2022-05-01

**Authors:** Samira Choudhury, Antonieta Medina-Lara, Richard Smith

**Affiliations:** a College of Medicine and Health, University of Exeter, St Luke's Campus, Heavitree Road, Exeter, EX1 2LU, England.

Before the coronavirus disease 2019 (COVID-19) pandemic, the World Health Organization (WHO) recognized antimicrobial resistance as one of the top 10 most urgent global health threats. Often referred to as the silent pandemic, antimicrobial resistance claims the lives of over 700 000 people annually. A study suggests that if no actions are taken, antimicrobial resistance will cause 10 million deaths per year by 2050 and an economic impact of over 100 trillion United States dollars.^1^ Efforts to tackle antimicrobial resistance as a critical global health concern have only recently intensified. Between 2017 and 2019, participation in the WHO Global Antimicrobial Resistance and Use Surveillance System increased exponentially, aggregating data from more than 64 000 surveillance sites from 66 countries.^2^ In August 2020, 94 countries were enrolled in this surveillance system.^3^ This high participation is a significant achievement in the global fight against this health threat. However, increasing concerns exist that the COVID-19 pandemic has set back the current and future progress against antimicrobial resistance.^4^ The pandemic has placed an enormous pressure on health-care systems and diverted resources, personnel and attention from the diagnosis and management of antimicrobial resistance to COVID-19 diagnosis and contact tracking and tracing. Antimicrobial resistance research has been severely disrupted and surveillance and antimicrobial stewardship programmes have also been deprioritized, delayed or halted.^5^

Nevertheless, the COVID-19 pandemic has highlighted the vulnerability of health-care systems in controlling infectious disease threats and increased awareness of the importance of planning for emerging infections and maintaining robust infection control. The pandemic has generated opportunities that should be seized to harness positive effects on the management of antimicrobial resistance. At least five such opportunities exist. 

First, COVID-19 has had a significant influence on our social interactions. People are now far more conscious of preventive health-care measures such as regular handwashing, wearing face masks and observing physical distancing.^6^ These behaviour changes will help prevent transmission of infectious diseases, including those affected by antimicrobial resistance; however, a risk of recidivism exists once the pandemic subsides.^7^

Second, COVID-19 has increased awareness of the importance of surveillance and laboratory capacity.^8^ Robust diagnostic and laboratory surveillance systems are crucial components of the COVID-19 mitigation response. Re-purposing this capacity towards antimicrobial resistance will be effective since these components are needed to provide accurate diagnosis of infectious diseases and assess the performance of antimicrobial stewardship programmes within the health-care system, especially in low- and middle-income countries.^9^ Simultaneously, the willingness of health-care authorities to invest in other diagnostics such as rapid bacterial infectious disease and point-of-care testing to tackle COVID-19 can be applied for antimicrobial resistance.^10^ This investment may encourage health-care authorities to emulate infrastructure that has rapidly been assembled such as lateral flow tests and polymerase chain reaction tests for the testing and diagnosis of COVID-19, at scale, for the detection of antimicrobial resistance.

Third, the COVID-19 pandemic has illustrated the importance of improving infection prevention and hygiene and is a reminder that compliance with infection prevention and control protocols is critical in minimizing the risk of hospitalizations. These protocols, which are necessary to prevent the transmission of severe acute respiratory syndrome coronavirus 2 (SARS-CoV-2), may substantially reduce antimicrobial resistance prevalence.^8^

Fourth, COVID-19 has facilitated wider awareness and reinforced the role of a global One Health approach. The application of the One Health approach has the potential to effectively combat COVID-19,^11^ and this approach can be leveraged to tackle the rise of antimicrobial resistance.

Finally, vaccines that effectively protect against SARS-CoV-2 will help reduce the global prevalence of COVID-19 and reduce the inappropriate use of antibiotics, thereby potentially lowering the burden of antimicrobial resistance. Promoting vaccine use for preventable diseases can have a substantial impact on the transmission of antimicrobial resistance.^12^

We need to ensure that one of the legacies of COVID-19 is that more support is given to antimicrobial resistance research, implementation of diagnostics, appropriate diagnostic stewardship and strengthening our health systems. A major opportunity exists as we emerge from the pandemic for the health community to increase collaboration with policy-makers, the public and the media, to raise antimicrobial resistance awareness and to increase engagement to build on the COVID-19 legacy of physical distancing, handwashing, face-coverings, vaccine development and of avoiding unnecessary use of antibiotics. Countries will need to increase efforts to work as a multidisciplinary community and combat antimicrobial resistance collectively. Multisectoral, coordinated and targeted research on such resistance and in line with the One Health approach will be critical for an effective containment of antimicrobial resistance in the context of the pandemic and will help present opportunities for tackling antimicrobial resistance.
